# MetaDP: a comprehensive web server for disease prediction of 16S rRNA metagenomic datasets

**DOI:** 10.1007/s41048-016-0033-4

**Published:** 2017-01-10

**Authors:** Xilin Xu, Aiping Wu, Xinlei Zhang, Mingming Su, Taijiao Jiang, Zhe-Ming Yuan

**Affiliations:** 1 Hunan Provincial Key Laboratory for Biology and Control of Plant Diseases and Insect Pests, Hunan Agricultural University, Changsha, 410128 China; 2 Center for Systems Medicine, Institute of Basic Medical Sciences, Chinese Academy of Medical Sciences & Peking Union Medical College, Beijing 100005; Suzhou Institute of Systems Medicine, Suzhou, 215123 China; 3 Suzhou Geneworks Technology Company Limited, Suzhou, 215123 China; 4 Institute of Basic Medical Sciences, Chinese Academy of Medical Sciences & Peking Union Medical College, Beijing, 100005 China

**Keywords:** Disease prediction, 16S rRNA, Metagenomics, Intestinal bowel syndrome

## Abstract

High-throughput sequencing-based metagenomics has garnered considerable interest in recent years. Numerous methods and tools have been developed for the analysis of metagenomic data. However, it is still a daunting task to install a large number of tools and complete a complicated analysis, especially for researchers with minimal bioinformatics backgrounds. To address this problem, we constructed an automated software named MetaDP for 16S rRNA sequencing data analysis, including data quality control, operational taxonomic unit clustering, diversity analysis, and disease risk prediction modeling. Furthermore, a support vector machine-based prediction model for intestinal bowel syndrome (IBS) was built by applying MetaDP to microbial 16S sequencing data from 108 children. The success of the IBS prediction model suggests that the platform may also be applied to other diseases related to gut microbes, such as obesity, metabolic syndrome, or intestinal cancer, among others (http://metadp.cn:7001/).

## Introduction

A wide variety of microbes live in the human body. These microbes exist in oral, nasopharynx, skin, gut, and many other regions of the body and play an important role in human health (Human Microbiome Project 2012; Sankar et al. 2015). To date, there is still significant uncertainty about the relationships between resident microbes and human diseases.

Most microorganisms in the human body have remained uncultured. Therefore, traditional methods for the inspection and identification of the microbial species have significant limitations. In 1998, Handelsman et al. first put forward the concept of the “metagenome” (Handelsman et al. 1998), and defined it as the genes and genomes of all of the microorganisms in an environmental sample. With the rapid development of high-throughput sequencing technology and the establishment of numerous microbial databases, metagenomics has become an emerging topic of interest in biomedical research. Recently, multiple metagenomics studies have revealed that microbial communities are associated with human diseases. Turnbaugh et al. characterized the gut microbial communities of 154 individuals and found that obesity was associated with phylum-level change in the microbiota and reduction of bacterial diversity (Turnbaugh et al. 2009). Pushalkar et al. studied five saliva microbial samples and found fifteen unique phylotypes in three oral squamous cell carcinoma subjects (Pushalkar et al. 2011). The relationships between microorganisms and some other diseases have also been investigated, such as oral diseases (Belda-Ferre et al. 2012), neurological diseases (Hsiao et al. 2013), rheumatoid arthritis (Scher et al. 2013), and Crohn’s disease (Gevers et al. 2014). Furthermore, some computational models have been constructed for disease classification and prediction based on metagenomic data. Qin et al. analyzed the differences between type 2 diabetes (T2D) patients and non-diabetic controls in 345 Chinese gut microbial samples. The researchers chose 50 gene markers to develop a T2D classifier model and used it for risk assessment and monitoring of T2D (Qin et al. 2012). Saulnier et al. compared the gut microbiomes of healthy children and pediatric patients with irritable bowel syndrome (IBS), and found some differences in the microbial communities in these two sample sets, which might suggest a novel technique for the diagnosis of pediatric patients with functional bowel disorders (Saulnier et al. 2011). Moreover, Qin et al. developed a support vector machine (SVM) model and indicated that microbiota-targeted biomarkers may serve as new tools for disease diagnoses (Qin et al. 2014). These prediction models indicate that metagenomics data can perhaps play an important role in the prevention and early diagnosis of disease.

Although numerous tools and methods have been developed to investigate the relationship between microbes and human diseases, there is still an absence of a general automated workflow from raw data to disease prediction. Some metagenomic data analysis tools, such as QIIME (Caporaso et al. 2010a, b), mother (Schloss et al. 2009), and RDP classifier (Wang et al. 2007), are readily amenable to running automated analyses, especially for biologists with minimal bioinformatics backgrounds. To address this problem, we developed a web-based platform called MetaDP, in which an automated analysis workflow was built for 16S rRNA sequences generated by both the 454 and Illumina platforms. The web server is constructed based on the open-source bioinformatics platform, Galaxy (Goecks et al. 2010) (https://galaxyproject.org/). In MetaDP, we integrated a number of metagenomics-associated tools and further built an automatic analysis pipeline. MetaDP also presents a user-friendly interface for one-stop automatic analysis and provides most of the output results in downloadable figure formats.

Previously reported 16S rRNA sequencing data from IBS disease were imported into the MetaDP platform. Based on microbial information from pediatric patients with IBS and healthy children, we constructed an IBS disease prediction model with a high degree of accuracy. This model is integrated into the MetaDP platform and may be helpful for IBS prevention and early diagnosis. The MetaDP web server is available publically (http://metadp.cn:7001/).

## Results

### The MetaDP framework

**Fig. 1:**
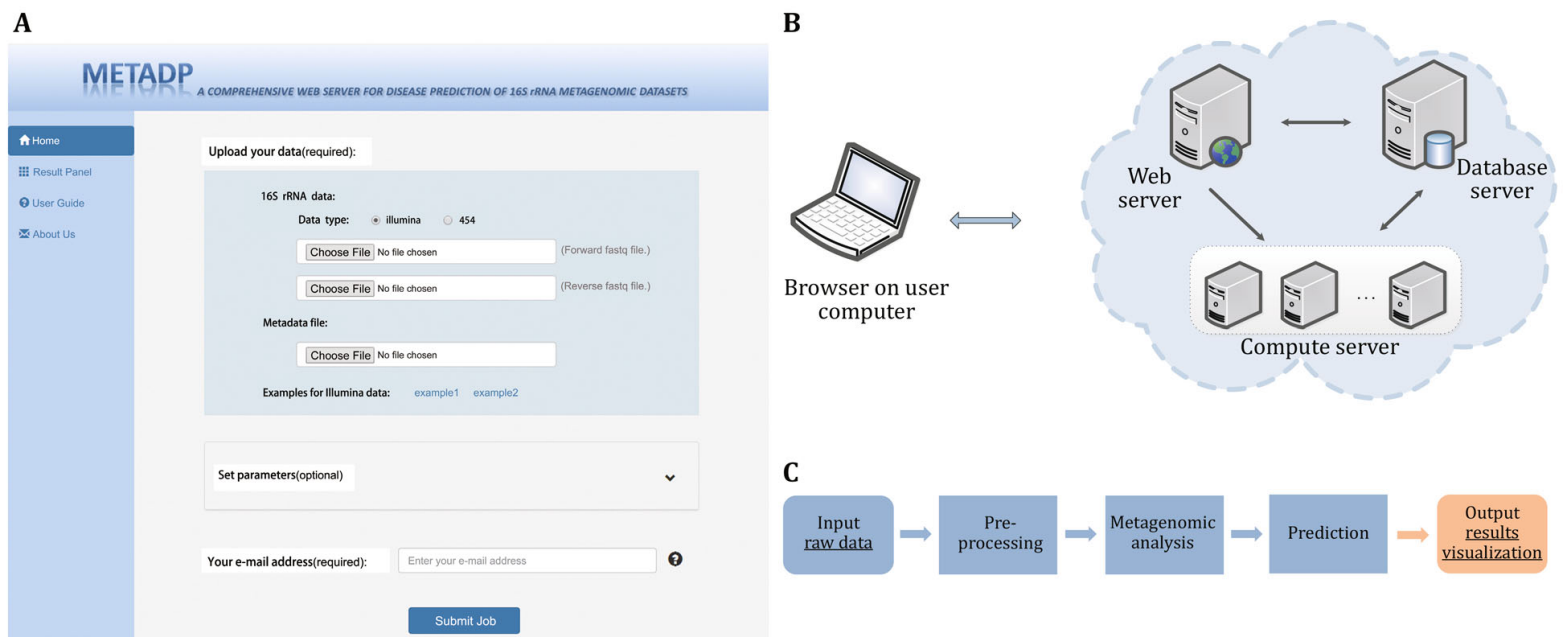
The framework of MetaDP. **A** User interface of web server. **B** System architecture. **C** Main steps of analysis

The MetaDP webserver is freely available at (http://metadp.cn:7001/) (Fig. 1A, B). MetaDP provides pre-defined workflows and can be used without registration. It begins with a straightforward process whereby a user uploads sequencing data. The analysis mainly includes three parts: data pre-processing, traditional metagenomic data analysis, and disease prediction (Fig. 1C). Pre-processing includes filtering low-quality sequences, splitting libraries based on the barcodes, removing chimeric sequences, and assembling reads. Traditional metagenomic data analysis includes microbial composition taxonomic analysis, alpha diversity, and beta diversity. The disease prediction aspect classifies testing samples with our pre-defined disease prediction model. The essential purpose of the MetaDP web service is to provide a user-friendly automated analysis system, in which users simply upload their raw data generated from a high-throughput sequencing platform. Thereby, the MetaDP may be readily used. There is no need for installing, integrating, and designing individual tools. In addition, MetaDP provides some optional parameters for better analysis.

### Metagenomic data analysis

#### Operational taxonomic unit (OTU) counting

For our dataset, after the pre-processing step, filtered sequences were clustered by the Uclust method (with a sequence similarity threshold of 97%). Then, the longest sequence from each cluster was chosen as its representative sequence. The OTU summary (http://metadp.cn:7001/metadp/F1/OTU_summary.txt) included 91,470 OTUs in a total of 2,448,155 sequence counts, in which the maximal OTU count among samples was 76,939. The microbial composition summary for each taxonomic level (from phylum to genus) is shown in Table 1.

**Table 1:** The counts of microbial communities at different taxonomy levels

Level	Counts
Phylum	14
Class	25
Order	42
Family	82
Genus	169

#### Taxonomic abundance

**Fig. 2:**
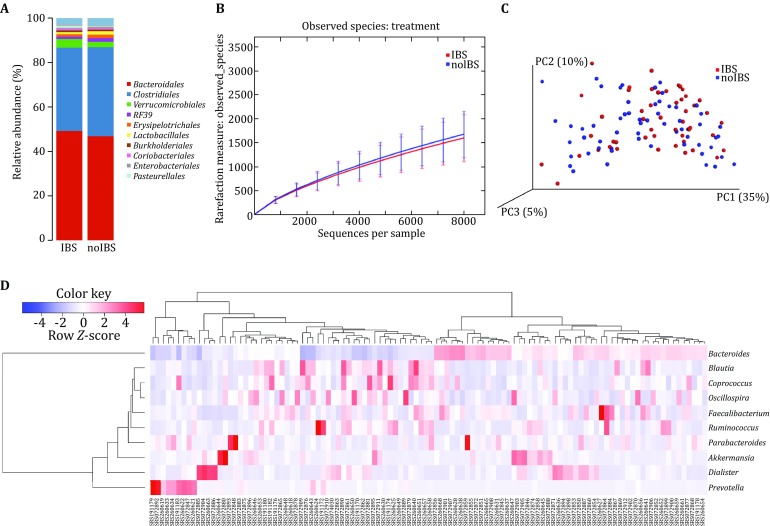
Visualization results of metagenomic data analysis. **A** Taxonomic abundance comparison between children with IBS and healthy children. **B** Rarefaction curve by groups. **C** Weighted UniFrac PCoA. The IBS and healthy samples are colored as *red* and *blue*, respectively. **D** Heatmap analysis for the top ten microbes at the genus level. Microbes and samples are both clustered. *Each row* is scaled by *Z*-score

Taxonomic binning of classified sequences was generated at five levels, from phylum to genus (http://metadp.cn:7001/metadp/F2/barchart_for_samples.html). Samples were grouped and averaged to plot the stacked bar charts (http://metadp.cn:7001/metadp/F3/barchart_for_groups.html). Another group of stacked bar plots were generated with the sorted taxonomic abundance data in samples (http://metadp.cn:7001/metadp/F4/OTU_sorted_barplot_for_samples.pdf) and in groups (http://metadp.cn:7001/metadp/F5/OTU_sorted_barplot_for_groups.pdf). Figure 2A shows the microbial stacked bar plot for the grouped sorted data of IBS versus noIBS samples at the order level. The analysis indicated that there is no obvious difference between the two groups, and the main microbes of these two groups are all *Bacteroidales* and *Clostridiales*, which is consistent with previously reported results (Riehle et al. 2012; Saulnier et al. 2011).

#### Alpha diversity

Alpha diversity analysis provides insight into differences in species abundance, richness, and evenness. Alpha diversity indices were analyzed with the default metrics, Chao1, ACE, Simpson, Shannon, Good’s coverage, and PD whole tree (http://metadp.cn:7001/metadp/F6/alpha_index_table.txt). Plots were generated and exported for rank-abundance, rarefaction index, and species richness. The rank-abundance curve (http://metadp.cn:7001/metadp/F7/rank_abundance_plot.pdf) is a 2D chart with abundance rank on the *X*-axis and relative abundance on the *Y*-axis. The alpha rarefaction analysis was performed by computation with multiple metrics (defaults: chao1, Shannon, and observed species) (http://metadp.cn:7001/metadp/F8/alpha_rarefaction_plot.html). Figure 2B shows the rarefaction curve displayed by groups that were analyzed with the observed species metrics. This curve demonstrates that the number of species in the two groups increased gradually with increasing sample sequence number, eventually saturating. The curve also indicates that the species richness of the noIBS sample (the blue line) is higher than that of the IBS sample (the red line).

#### Beta diversity

Beta diversity analysis provides a measure of the distance between each sample. Both weighted and unweighted distance matrices were calculated and visualized with Principal coordinates analysis (PcoA) plots (http://metadp.cn:7001/metadp/F9/weighted_PCoA.html and http://metadp.cn:7001/metadp/F10/unweighted_PCoA.html). Figure 2C shows the weighted-distance distribution of samples in 3D space. In this figure, both IBS (red) and noIBS (blue) samples are mixed, indicating that it was difficult to classify the samples according to the distance matrix.

#### OTU heatmaps

A heatmap was used to visualize the relationships between the OTUs and samples (http://metadp.cn:7001/metadp/F11/raw_OTUs_heatmap.html). In the heatmap, raw OTU counts per sample are displayed. Figure 2D presents the heatmap for the top 10 microbes at the genus level (other classification levels are listed in http://metadp.cn:7001/metadp/F12/top10_heatmaps.pdf). Both samples in columns and OTUs in rows were clustered by relative abundance, and the rows were scaled by *Z*-score.

### Prediction model

In total, 91,470 OTUs were obtained among 108 samples by OTU picking. After filtering for zero values (percentage >80% in all samples), 1726 OTUs were selected. Then, a *t* test was used to examine the discriminatory ability of each feature. Finally, 110 OTU feature sets were selected for the construction of the next model (http://metadp.cn:7001/metadp/F13/filtered_OTU_table.txt). The top 20 most significant features and their *p*-values are listed in Table 2. Within these features, *Bacteroides*, *Dorea,* and *Faecalibacterium* have been reported to be associated with IBS (Saulnier et al. 2011; Ghoshal et al. 2012; Rajilić-Stojanović et al. 2015).

**Table 2:** Information for the top 20 most significant features

OTU ID	*p*-value	Taxonomy
358944	0.001*	k_Bacteria;p_Firmicutes;c_Clostridia;o_Clostridiales;f_;g_;s_
New.CleanUp.ReferenceOTU327210	0.0014*	k_Bacteria;p_Bacteroidetes;c_Bacteroidia;o_Bacteroidales;f_Bacteroidaceae;g_Bacteroides;s_
189384	0.0034*	k_Bacteria;p_Bacteroidetes;c_Bacteroidia;o_Bacteroidales;f_Bacteroidaceae;g_Bacteroides;s_
199283	0.0034*	k_Bacteria;p_Firmicutes;c_Clostridia;o_Clostridiales;f_Ruminococcaceae;g_Faecalibacterium;s_prausnitzii
179665	0.0074*	k_Bacteria;p_Firmicutes;c_Clostridia;o_Clostridiales;f_Lachnospiraceae;g_Dorea;s_
New.ReferenceOTU288	0.0076*	k_Bacteria;p_Bacteroidetes;c_Bacteroidia;o_Bacteroidales;f_Rikenellaceae;g_;s_
New.CleanUp.ReferenceOTU389203	0.0077*	k_Bacteria;p_Firmicutes;c_Clostridia;o_Clostridiales;f_Lachnospiraceae;g_;s_
589277	0.0085*	k_Bacteria;p_Bacteroidetes;c_Bacteroidia;o_Bacteroidales;f_Bacteroidaceae;g_Bacteroides;s_
189855	0.0095*	k_Bacteria;p_Firmicutes;c_Clostridia;o_Clostridiales;f_Lachnospiraceae;g_;s_
187251	0.0103	k_Bacteria;p_Firmicutes;c_Clostridia;o_Clostridiales;f_Lachnospiraceae
New.ReferenceOTU7	0.0103	k_Bacteria;p_Bacteroidetes;c_Bacteroidia;o_Bacteroidales;f_Bacteroidaceae;g_Bacteroides;s_
2653002	0.0112	k_Bacteria;p_Bacteroidetes;c_Bacteroidia;o_Bacteroidales;f_Bacteroidaceae;g_Bacteroides;s_ovatus
New.CleanUp.ReferenceOTU262311	0.0114	k_Bacteria;p_Bacteroidetes;c_Bacteroidia;o_Bacteroidales;f_Bacteroidaceae;g_Bacteroides;s_
New.CleanUp.ReferenceOTU387907	0.013	k_Bacteria;p_Firmicutes;c_Clostridia;o_Clostridiales;f_Ruminococcaceae
New.ReferenceOTU412	0.0133	k_Bacteria;p_Bacteroidetes;c_Bacteroidia;o_Bacteroidales;f_Bacteroidaceae;g_Bacteroides;s_
New.ReferenceOTU156	0.0134	k_Bacteria;p_Bacteroidetes;c_Bacteroidia;o_Bacteroidales;f_Bacteroidaceae;g_Bacteroides;s_
196713	0.0136	k_Bacteria;p_Firmicutes;c_Clostridia;o_Clostridiales;f_Lachnospiraceae;g_;s_
1835779	0.0153	k_Bacteria;p_Firmicutes;c_Clostridia;o_Clostridiales;f_Lachnospiraceae
198990	0.0161	k_Bacteria;p_Firmicutes;c_Clostridia;o_Clostridiales;f_Lachnospiraceae;g_;s_
New.CleanUp.ReferenceOTU320393	0.0163	k_Bacteria;p_Bacteroidetes;c_Bacteroidia;o_Bacteroidales;f_Bacteroidaceae;g_Bacteroides;s_

^*^Represents extremely significant difference (*p* < 0.01)

**Fig. 3:**
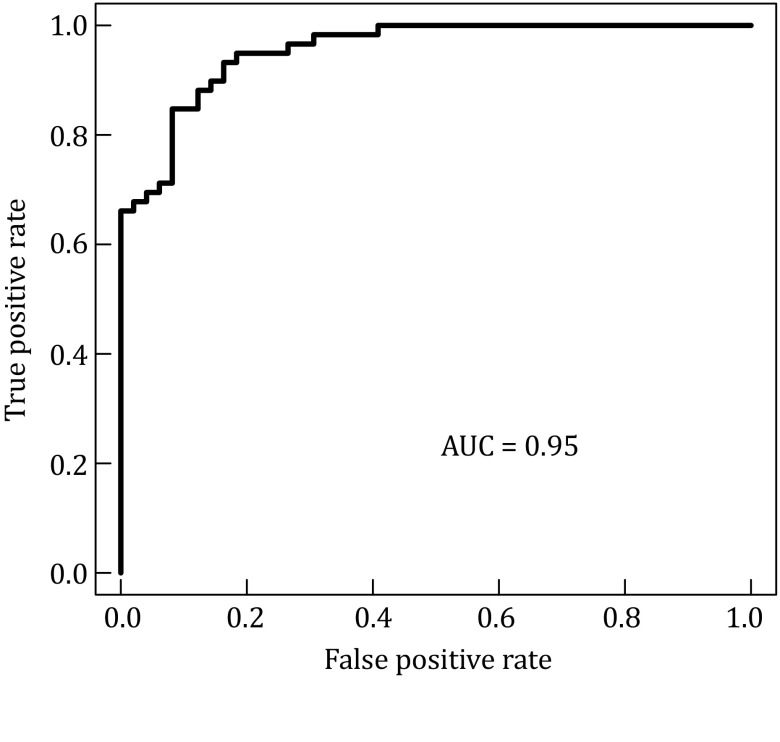
ROC curve of the SVM model of IBS disease. *X* and *Y* axes represent the false positive rates (1−sensitivity) and the true positive rates (sensitivity), respectively. The AUC score is 0.95

Then, the quantified feature vector could be input into LIBSVM. The radial basis function (RBF) kernel was used in LIBSVM, and a grid search program (grid.py) was used to obtain the optimized parameter combination *C* = 4.0, *γ* = 0.125. Thereby, the IBS prediction model was constructed successfully. To test the performance of the IBS model, tenfold cross-validation was adopted. The results show that the accuracy and the AUC score were 0.93 and 0.95, respectively (Fig. 3).

## Discussion

The MetaDP platform is a one-stop 16S rRNA sequencing data analysis flowchart with a friendly user interface that aims to help researchers investigate the structure and diversity of human microbial flora and provide deep insight into microorganisms associated with the disease. An automatic analysis workflow can be performed once users upload their raw sequencing data with barcodes. In this version, our platform provides a set of universal 16S rRNA data analysis tools to constitute a workflow for data from the 454 and Illumina platforms. The workflow outputs the bacterial distribution, alpha diversity, beta diversity, and disease risk assessment with a plug-in prediction model. To build the prediction model, we used IBS as an example with a total of 108 microbial samples. In the near future, we will increase the sample size of intestinal microbial diseases and improve the prediction model.

In future work, MetaDP will provide an open API interface, so that researchers can easily integrate other bioinformatics tools and data analysis workflows with our platform. We will also integrate more metagenomic data analysis tools, data analysis workflows, and machine learning models, making our platform useful for the analysis of more diseases. Users can also perform custom/personalized data analysis processes according to their own requirements. The MetaDP platform can be easily used for microorganism-associated diseases, such as diabetes, obesity, and colorectal cancer, among others. We will collect more intestinal microbial sequencing data to expand disease prediction models for better disease prevention and diagnosis.

## Materials and methods

**Fig. 4:**
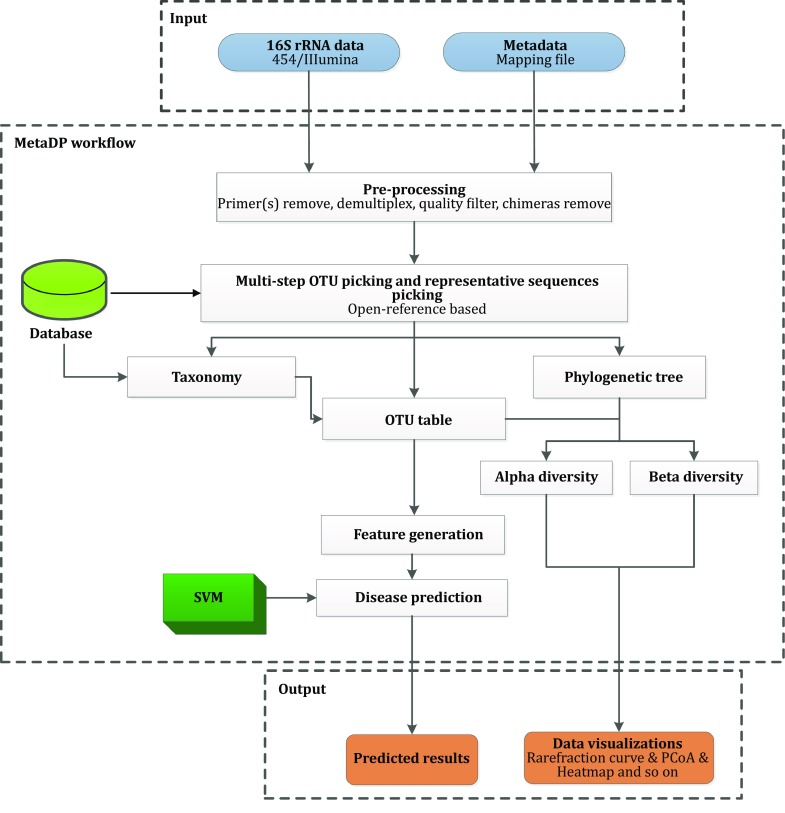
Overview of the MetaDP workflow for 16S rRNA sequences analysis and disease prediction. The workflow supports the input of 16S rRNA sequencing data and sample metadata. The analysis includes sequence pre-processing, OTU picking, biodiversity analysis, and disease prediction with the configured SVM model. Predicted results and visualized data are returned

MetaDP provides pre-defined workflows for metagenomic data analysis and disease prediction modeling based on the Galaxy platform (Fig. 4). Users simply need to upload their raw 16S sequencing data generated by 454 pyrosequencing or by the Illumina platform and another metadata mapping file with detailed sample information, including sample names, barcodes, descriptions of the columns. The core analysis pipeline consists of demultiplexing, quality filtering, OTU picking, taxonomic assignment, phylogenetic reconstruction, diversity analysis, and visualization. In addition, a configured SVM-based prediction model has been constructed for intestinal bowel syndrome.

### Data pre-processing

First, sample isolation and quality control must be performed from multiplexed Standard Flowgram Format (SFF) file or FASTQ files. The four main steps for raw data pre-processing are as follows. (1) Sample demultiplexing: the multiplexed reads are assigned to samples based on their unique nucleotide barcodes in the mapping file. (2) Primer removal: during demultiplexing, the primer sequences and barcodes have to be removed at the same time. (3) Quality filtering: short or low average quality score reads are removed using customized thresholds, and any sequence with the first nucleotide as “N”or “n” is cut. (4) Denoising and chimera removal: before sequence clustering, denoising and chimera removal are required for 454 and Illumina datasets. In this platform, chimera detection is based on the USEARCH 6.1 algorithm (Edgar 2010). The above steps are all run by calling QIIME (Caporaso et al. 2010a, b). Paired-end reads for the Illumina platform are trimmed using Trimmomatic (Bolger et al. 2014). Then, FLASH software (Magoc and Salzberg 2011) is used to assemble the trimmed paired-end reads, and the resulting contigs are compiled into an input file to use for the next sample demultiplexing step.

### Metagenomic data analysis

#### OTU picking

OTUs are normally used for analyzing microbial composition and diversity. Pre-processing sequences are grouped into a cluster when their sequence similarities are greater than the threshold value, such as 97% at the species level. In this study, we chose Uclust (Edgar 2010) as the default OTU clustering tool. The five steps for OTU picking are given as follows. (1) Pre-filtering: the sequences are searched against the GreenGenes reference database (DeSantis et al. 2006), filtered for at least a low percent identity (default: 0.60), and discarded if they fail to match. (2) Multi-step OTU picking: the pre-filtered sequences are aligned with an existing database, and added to the database as new reference sequences if the sequences are mismatched. (3) Representative sequence picking: the longest sequence is chosen as the representative sequence. (4) Taxonomic assignment: a taxonomic classification is assigned to each sequence of the representative set with the GreenGenes database and newly defined taxonomies from step 2. (5) OTU table generation: an OTU table is constructed in the Genomics Standards Consortium candidate standard Biological Observation Matrix (BIOM) format. The BIOM format file can be converted to other formats with a series of scripts available from the BIOM project (McDonald et al. 2012).

#### Phylogenetic analysis

Representative sequences are assigned to the core set of the GreenGenes database (DeSantis et al. 2006) with PyNAST (Caporaso et al. 2010a, b). Then, the sequence alignment is filtered by removing the gap regions from every sequence. The FastTree method (Price et al. 2009) is utilized to construct phylogenetic trees based on the filtered sequence files. The phylogenetic tree can be interactively displayed through an online tool named Interactive Tree of Life (iTOL, http://itol.embl.de/) (Ciccarelli et al. 2006).

#### Taxa summaries

A taxa summary summarizes the relative abundance of different taxonomic levels (from phylum to genus) among all samples based on an OTU table. Sequences are taxonomically binned based on the output of a local copy of the ribosomal database project (RDP) classifier. Normalized data are produced from the relative abundances of taxa present in each sample. Any unclear taxa are combined and named “other.” The results from the taxonomic binning of classified sequences are displayed as bar charts, which make it easier to convey the main compositions of the samples.

#### Biodiversity

Two types of diversity measurements (alpha diversity and beta diversity) are usually used for assessing the relatedness of metadata attributes on OTU tables. Alpha diversity is mainly used to estimate the diversity of a microbial community within a group of samples, through a series of statistical indices such as Chao1, ACE, Shannon, Simpson, Good’s coverage, and so on (Navas-Molina et al. 2013). Rarefaction curves are plotted by counting the OTU numbers from random reads of the samples based on these diversity metrics. Beta diversity is mainly used to compare the differences of microbial communities between samples. UniFrac (Lozupone et al. 2011) is always used for comparing biological communities. Both weighted and unweighted variants of UniFrac are widely used. The former accounts for the abundance of OTUs, while the latter only considers their presence or absence. The distance metrics are investigated through PCoA, and an interactive 3D plot is generated.

#### OTU heatmaps

For the composition analysis of OTUs among samples, two types of OTU heatmaps are provided. The first type of heatmap is an interactive plot and is directly colored to reflect the absolute abundance of raw OTUs. The other type of heatmap is a bi-directional map, in which both the samples and the taxa summary are clustered. Users can set the threshold for the microbes at different classification levels (the default is top ten microbes at the genus level).

### Disease prediction model

#### Feature selection

Feature selection (Saeys et al. 2007), also known as variable selection or attribute selection in machine learning, is the selection of a subset of redundancy features for the construction of a prediction model. In this study, feature selection is used mainly for the simplification of models for better feature interpretation, and for the reduction of overfitting. In our training set, the values of the OTU tables generated in the metagenomics data analysis were used. For each feature, a value with zero is deleted first, then feature selection is performed based on the statistical comparison.

#### Support vector machine (SVM)

SVMs are important supervised learning algorithms for classification and regression analysis. In recent years, SVMs have been widely used in life sciences research, such as for studies on alternative splice site recognition, biomarker selection, remote homology detection, gene function prediction, and protein–protein interaction prediction, among others (Pavlidis et al. 2002; Liao and Noble 2003; Ben-Hur and Noble 2005; Ratsch et al. 2005; Sonnenburg et al. 2007). Some useful software packages have also been developed (Bottou 2007; Fan et al. 2008; Chang and Lin 2011). In this study, we use LIBSVM (Chang and Lin 2011) (http://www.csie.ntu.edu.tw/~cjlin/libsvm), which is an integrated software package for support vector classification, regression, and distribution estimation. An SVM can efficiently perform a non-linear classification through a so-called kernel function, thus implicitly mapping inputs into high-dimensional feature spaces. The RBF kernel was chosen for our study. The penalty parameter *C* and kernel parameters *γ* in the RBF kernel are optimized to result in the best prediction performance. To obtain the optimal *C* and *γ* values, we used the grid search method. The main steps for a grid search can be described as follows. First, *M* and *N* numbers of *C* and *γ* values are assigned, respectively. Then, different SVM models with *M* × *N* (*C*, *γ*) numbers of parameters combined are trained. Finally, the optimal pair of parameters is selected.

#### Evaluation

Cross-validation (tenfold) is used to estimate the performance of our prediction model. In this study, we use SVM-train with parameter –*v* 10, as it will randomly split samples into ten subsamples; each subsample is used once as the validation data for testing the model, and the remaining nine subsamples are used as training data; finally, the average accuracy will be reported. A receiver-operating characteristic (ROC) curve is used to illustrate the performance of the classifier model. The ROC curve plots the true positive rate (TPR) against the false positive rate (FPR) at various threshold values. The TPR and FPR are given by *TPR* = *TP*/(*TP* + *FN*) and *FPR* = *FP*/(*FP* + *TN*), respectively. The area under the ROC curve (AUC) score is used to estimate the overall classifier performance. The ROCR package from CRAN (http://cran.r-project.org/) was used to calculate the TPR and FPR values and to draw ROC curves, the AUC scores were also provided to estimate this classifier model performance.

### Implementation

MetaDP has been implemented in a local Galaxy instance running under a GNU/Linux operating system. Galaxy was obtained from http://wiki.galaxyproject.org/Admin/GetGalaxy and intentionally installed as a normal user (“galaxy”) for easy migration and security. The advantage of using the Galaxy framework for MetaDP is that Galaxy provides a web-accessible platform to integrate different command-line tools and has a customized workflow configuration system. Additionally, Galaxy provides some useful functional dependencies, such as a web service (Nginx), database storage (MySQL), a job queuing system, and history management. In MetaDP, we integrated the metagenomic data analysis package QIIME, the SVM model, and NGS quality control tools. The applications of all tools were implemented with XML files, Python, Perl, and Shell wrappers. These tools consisted of the specific workflow for library splitting, OTU picking, taxonomy analysis, rarefaction analysis, and disease prediction. For ease of use, we simplified the operations of the web applications and designed a more interactive and user-friendly website. The user simply needs to upload input files and run the workflow through a web interface.

### Datasets

In total, 108 samples (49 samples from pediatric patients with IBS and another 59 samples from healthy children) of 16S rRNA 454 sequencing data were downloaded from the NCBI database (http://www.ncbi.nlm.nih.gov/sra, SRP002457) (Saulnier et al. 2011).

## Abbreviation

MetaDP: Disease prediction of metagenomic datasets
